# (R)-Roscovitine and CFTR modulators enhance killing of multi-drug resistant *Burkholderia cenocepacia* by cystic fibrosis macrophages

**DOI:** 10.1038/s41598-020-78817-x

**Published:** 2020-12-10

**Authors:** Chandra L. Shrestha, Shuzhong Zhang, Benjamin Wisniewski, Stephanie Häfner, Jonathan Elie, Laurent Meijer, Benjamin T. Kopp

**Affiliations:** 1 Division of Pulmonary Medicine, Center for Microbial Pathogenesis, The Abigail Wexner Research Institute At Nationwide Children’s Hospital, 700 Children’s Drive, Columbus, OH 43205 USA; 2 Rudolf-Boehm-Institut F. Pharmakologie U. Toxikologie Medizinische Fakultät, Universität Leipzig, Leipzig, Germany; 3 ManRos Therapeutics, Perharidy Peninsula, Roscoff, France

**Keywords:** Monocytes and macrophages, Cystic fibrosis, Target validation

## Abstract

Cystic fibrosis (CF) is characterized by chronic bacterial infections and heightened inflammation. Widespread ineffective antibiotic use has led to increased isolation of drug resistant bacterial strains from respiratory samples. (R)-roscovitine (Seliciclib) is a unique drug that has many benefits in CF studies. We sought to determine roscovitine’s impact on macrophage function and killing of multi-drug resistant bacteria. Human blood monocytes were isolated from CF (F508del/F508del) and non-CF persons and derived into macrophages (MDMs). MDMs were infected with CF clinical isolates of *B. cenocepacia* and *P. aeruginosa.* MDMs were treated with (R)-roscovitine or its main hepatic metabolite (M3). Macrophage responses to infection and subsequent treatment were determined. (R)-roscovitine and M3 significantly increased killing of *B. cenocepacia* and *P. aeruginosa* in CF MDMs in a dose-dependent manner. (R)-roscovitine-mediated effects were partially dependent on CFTR and the TRPC6 channel. (R)-roscovitine-mediated killing of *B. cenocepacia* was enhanced by combination with the CFTR modulator tezacaftor/ivacaftor and/or the alternative CFTR modulator cysteamine. (R)-roscovitine also increased MDM CFTR function compared to tezacaftor/ivacaftor treatment alone. (R)-roscovitine increases CF macrophage-mediated killing of antibiotic-resistant bacteria. (R)-roscovitine also enhances other macrophage functions including CFTR-mediated ion efflux. Effects of (R)-roscovitine are greatest when combined with CFTR modulators or cysteamine, justifying further clinical testing of (R)-roscovitine or optimized derivatives.

## Introduction

**Figure 1:**
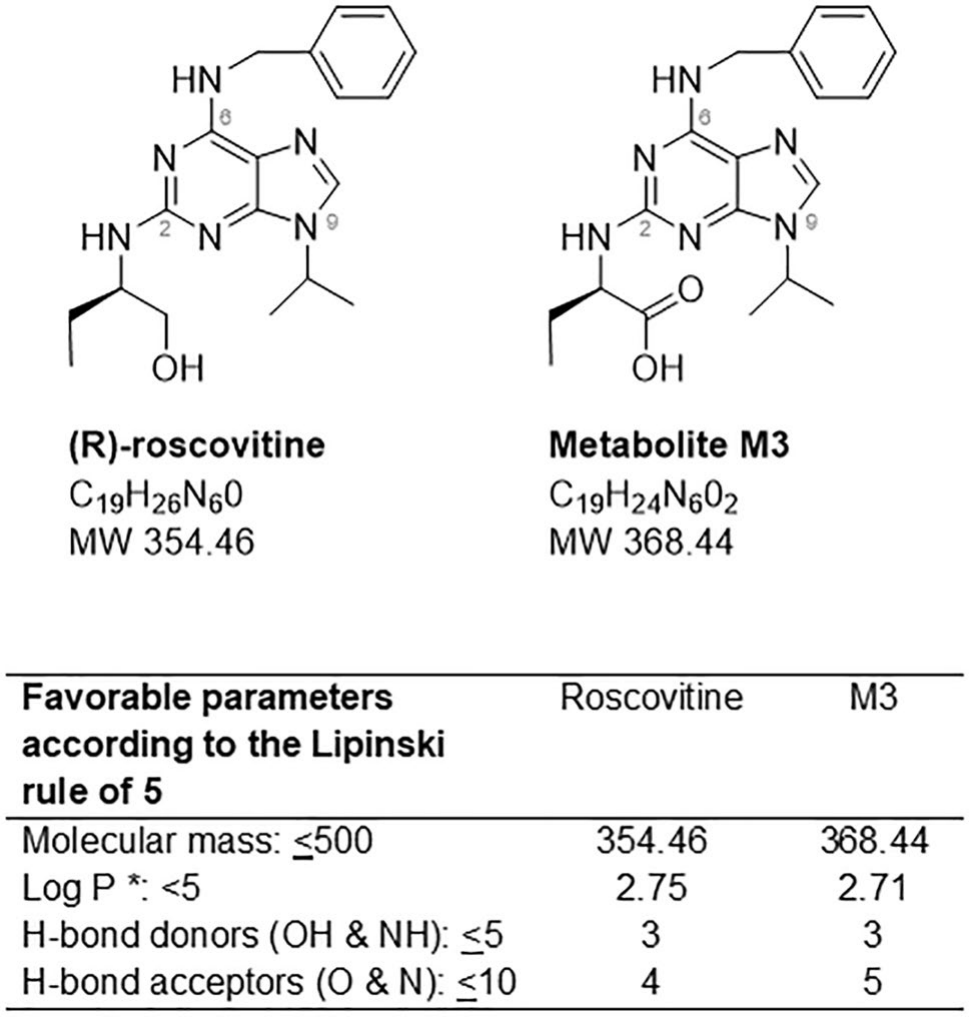
Structure of the 2,6,9-tri-substituted purine (R)-roscovitine and its main metabolite, M3. Drugability of both compounds according to the Lipinski rule of five. * P is the octanol–water partition coefficient.

Persons with cystic fibrosis (CF) remain plagued by chronic bacterial infections and inflammation-mediated tissue destruction. Of further concern is the growing incidence of multi-drug resistant bacterial strains isolated from CF respiratory samples^1, 2^. While new medications such as cystic fibrosis transmembrane conductance regulator (CFTR) modulators are improving outcomes in CF, their high cost^3, 4^ prohibits widespread use in many countries and in those without (sufficient) insurance. Further, although infections are reduced on CFTR modulator treatment ^5, 6^, chronic infection remains a consistent feature ^7^, indicating a continued need for novel approaches to the treatment of bacterial infections in CF.

(R)-roscovitine (Seliciclib, hereafter refered to as roscovitine) is a synthetic, low molecular weight, tri-substituted purine (Fig. 1), initially developed as a cyclin-dependent kinase inhibitor for the treatment of lung, breast, and nasopharyngeal cancers^8–11^. It has undergone many preclinical studies as well as phase 1 and phase 2 clinical trials in more than 520 patients, mostly as a potential anti-cancer drug. Roscovitine synthesis is a cheap, fast, optimized 3-step protocol^12^. The drug has good oral and lung availability, a short half-life, and the main hepatic metabolite (M3, Fig. 1) is inactive on kinases, but displays some other biological activities. Roscovitine has shown multiple benefits in CF studies^13–15^. Despite its lack of kinase inhibitory activity, the M3 metabolite of roscovitine is able, like roscovitine, to correct the intraphagolysosomal pH of alveolar macrophages and enhance their bactericidal activity^14^, and correct F508del-CFTR trafficking^15^. Recently, roscovitine was tested in the CF population in a Phase 2A trial ^16^ (ROSCO-CF: A Phase II, dose ranging, multicenter, double-blind, placebo controlled study to evaluate safety and effects of [R]-roscovitine in adults subjects with CF, with at least one F508del-CFTR mutation and chronically infected with *Pseudomonas aeruginosa*). We sought to build upon this trial and further determine the efficacy of roscovitine in CF, through examination of its impact upon restoration of macrophage function, including the ability to kill multi-drug resistant *Burkholderia cenocepacia.*

## Methods

### Subjects

People with a confirmed CF diagnosis and homozygous for the F508del mutation were recruited from the outpatient CF clinic at baseline health. People were excluded if using chronic immunosuppressants or if they had a history of transplantation. The diagnosis of CF was defined as two disease-causing mutations or a sweat chloride test ≥ 60 mmol/L. Human subject recruitment was approved by the Institutional Review Board of Nationwide Children’s Hospital (IRB15-00611). Informed consent and/or assent was obtained for all participants. All methods were performed in accordance with the Declaration of Helsinki for medical research involving human subjects. A parent or guardian of child participants provided informed consent on their behalf. Healthy, age and gender-matched controls were also recruited.

### Reagents

Roscovitine and M3 were synthesized at ManRos Therapeutics and used at concentrations ranging from 0.1 to 100 μM. TRPC6 inhibitor^17^ was provided by Dr. Stephanie Häfner and used at 10 µM. CFTR modulators tezacaftor and ivacaftor (Selleck chem) were used at 5 µM. Cysteamine (Sigma) was used at 5 µg/ml and the CFTR inhibitor Inh-172 (Sigma) was used at 5–10 µM. All reagents were dissolved in DMSO and therefore non-treatment conditions received equal DMSO concentrations. All reagents were given as pre-treatment for 6 h prior to general infection experiments, and 4 h prior to halide efflux and phagocytosis.

### Macrophage isolation

Heparinized blood samples were obtained from people with CF and non-CF healthy controls. Peripheral monocytes were isolated from whole blood using Lymphocyte Separation Medium (Corning, 25–072-CV). Isolated monocytes were re-suspended in RPMI (Gibco, 22400-089) plus 10% human AB serum (Corning, 35-060-Cl) and differentiated for 5 days at 37 °C into MDMs^18, 19^. MDMs were confirmed by microscopy and flow cytometry^20^. MDMs were then placed in a monolayer culture prior to infection.

### Bacterial strains and colony forming unit (CFU) assay

Macrophages were infected with RFP-expressing *B. cenocepacia* strain k56-2 or a multi-drug resistant *P. aeruginosa* isolate obtained from a CF patient’s sputum. The *B. cenocepacia* strain is representative of an epidemic clinical strain from the ET12 lineage^21^. Bacteria were reproducibly grown in LB media over 24 h. CFU analysis was performed as previously described^22^. Recovered bacteria were quantified by plating serial dilutions on LB agar plates and analyzed for CFUs.

### Direct bacterial killing

Direct bacterial killing assessment was performed per previous methods^23^. Briefly, bacteria were grown for 24 h and 4 × 10^8^ CFUs of bacteria were added in 200 µL of LB broth in 96-well plates with or without desired treatments. The control wells contained LB liquid media only. Serial dilution was performed up to 10^–7^ and roscovitine or M3 were added at concentrations from 0.1 to 100 µM at the start of incubation with bacteria as noted. For bacterial growth, serial dilutions were plated in LB agar plates and incubated for 24 h at 37 °C and CFUs were counted.

### Phagocytosis

Phagocytosis was performed per prior methods^20^. Briefly, RFP-expressing *B. cenocepacia* were fixed with 4% paraformaldehyde for 30 min at room temperature, washed with PBS 5 times, and re-suspended in PBS containing 10% AB serum for 60 min at 37 °C. Serum-opsonized *B. cenocepacia* were incubated with MDMs at 50:1 (bacteria to cells) for 40 min at 37 °C in the presence or absence of treatments as noted, and detached for FACS analysis after PBS washing.

### CFTR function

Cells were plated in a 96-well plate at 10^6^/100 μL/well and rested for 24 h. After 24 h media was removed and cells were washed with efflux solution (mM):135 NaNO_3_, 1 CaSO_4_, 1 MgSO_4_, 2.4 K_2_HPO_4_, 0.6 KH_2_PO_4_, ten HEPES and ten Glucose. A fluorescent indicator of intracellular Cl^−^ [*N*-Ethoxycarbonylmethyl-6-Methoxyquinolinium Bromide (MQAE), E3101, Thermofisher] was loaded with a hypotonic buffer added for 5 min at 37 °C in the dark. MQAE solution was then removed and cells were incubated in 100 μL/well of warmed halide efflux solution for 5 min at 37 °C. The maximum fluorescence was then recorded on a plate reader. The halide efflux solution was removed and intracellular MQAE quenched with warmed NaI buffer (135 mM, 100 μL/well) at 15 min at 37 °C. NaI buffer was removed and halide efflux solution added back for 5 min and basal fluorescence measured. Cells were then stimulated with forskolin (10 μM) and 3-Isobutyl-1-methylxanthine (IBMX,100 μM) or CFTRinh172 (10 μM) at 37 °C and fluorescence measured every 5 min for 30 min. The minimum fluorescence was obtained by treating the cells with the quenching buffer (150 mM KSCN, 5 μM valinomycin in NaI buffer) for 30 min at 37 °C to record the minimum fluorescence (autofluorescence). CFTR-dependent chloride efflux was calculated as the maximum fluorescence after forskolin stimulation.

### Immunoblotting

CFTR immunoblotting was performed as previously described^20, 24^. In brief, MDM supernatants were removed post treatment and the cells washed twice with PBS. MDMs were lysed in lysis buffer (10 mM Tris–HCl pH 7.8, 200 mM NaCl, 1 mM EDTA, 0.5% Sodium Deoxycholate, 1% NP-40) with a protease inhibitor (Roche Applied Science, 10-519-978-001). Then 30 µg of protein was denatured in Laemmli sample buffer for 10 min at 95 °C, and then separated by SDS-PAGE and transferred onto polyvinylidene difluoride membranes. Membranes were immunoblotted for anti-CFTR (anti-CFTR 596, obtained from the CFTR Antibody Distribution Program https://www.cff.org/Research/Researcher-Resources/Tools-and-Resources/CFTR-Antibodies-Distribution-Program/) or GAPDH (2118, Cell Signaling). Protein bands were detected with HRP-conjugated secondary antibodies and visualized using Pierce ECL reagents (Life Sciences, Thermofisher, 32106). Bands were quantified by densitometry and analyzed using ImageJ.

### Statistics

Statistical analyses were performed using GraphPad Prism software (version 7.05). Two sample unpaired t-tests were used for independent sample comparisons. One-way ANOVA with post-hoc Tukey tests were used for multiple comparisons. Statistical significance was defined as a p value < 0.05.

### Conference presentation

Portions of this manuscript were presented at the 2019 North American Cystic Fibrosis Conference.

## Results

### Patient demographics

The demographic characteristics of the persons who donated blood for the study are listed in Table 1**.** The CF donors were relatively homogenous, with a targeted *Phe508del* homozygous population to avoid genotype confounders. Of note, approximately 40% of CF patients were on a CFTR modulator at blood draw (lumacaftor/ivacaftor or tezacaftor/ivacaftor). This was consistent with our overall clinic population during the study timeframe.

**Table 1:** Cohort demographics.

	CF	Non-CF	P value
Subjects (n)	32	32	–
Female	43.8%	53.1%	0.46
Age (years)	28.6 ± 13.8	33.6 ± 8.1	0.09
Caucasian	100%	100%	–
*Phe508del* homozygous	100%	–	–
CFTR modulator use	40.6%	–	–

Values as expressed as percentage or means ± standard deviation.

### Roscovitine increases CF macrophage-mediated killing of multi-drug resistant bacteria

**Figure 2:**
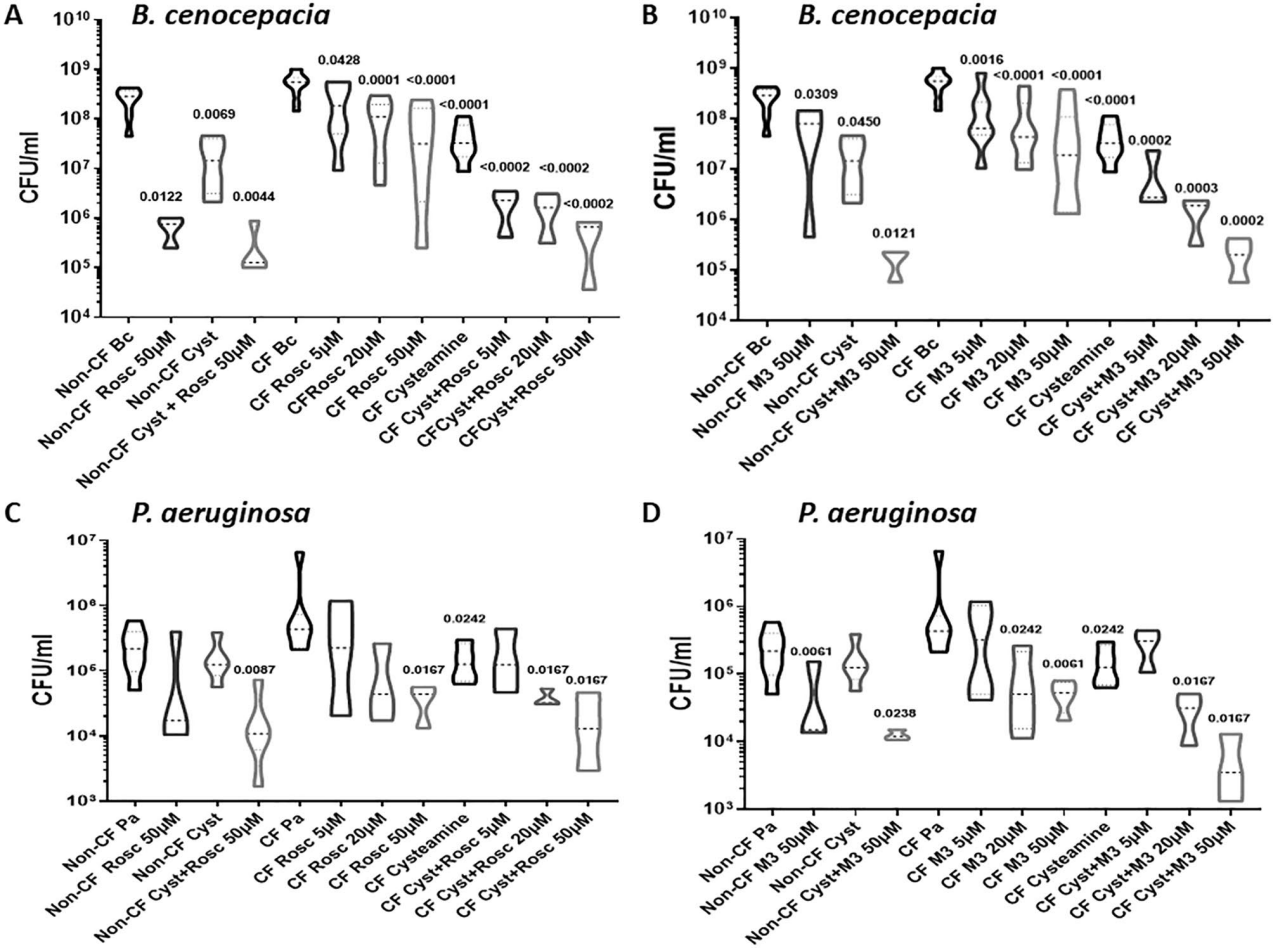
Roscovitine and M3 metabolite increase CF macrophage-mediated killing of multi-drug resistant bacteria. CFU assay for CF MDMs infected with multi-drug resistant clinical isolates of *B. cenocepacia* (Bc, **A**,**B**) and *P. aeruginosa* (Pa, **C,D**). CF MDMs were grouped according to the presence or absence of treatment with increasing concentrations of roscovitine **(A,C)**, M3 **(B,D)**, or cysteamine **(A–D)**. Data represented as violin plots to show distribution of data. Dose ranges of roscovitine and M3 shown only for CF. n = 3–8/group, one-way ANOVA with post-hoc Tukey. Group ANOVA p value < 0.0001 for *B. cenocepacia* group. Group ANOVA p value 0.6832 for *P. aeruginosa* group. Individual p values shown for comparisons between non-CF columns with non-CF Bc or non-CF Pa as reference, and between CF columns with CF Bc or CF Pa as reference.

Roscovitine and M3, its main hepatic carboxylated metabolite^25, 26^, are low molecular weight, 2,6,9-trisubstituted purines which match the Lipinski “rule of five” which defines molecules with a high drugability potential (Fig. 1)^27^. To determine the impact of roscovitine on macrophage-mediated killing of *B. cenocepacia*, we isolated MDMs from CF and non-CF donors and infected them with *B. cenocepacia* prior to treatment with increasing concentrations of roscovitine or M3. Both roscovitine (Fig. 2A) and M3 (Fig. 2B) demonstrated a significant reduction in bacterial load in a dose-dependent manner, with greater than one log reduction in bacterial load observed at 50 μM. Concentrations beyond 50 μM were associated with increased cell death (data not shown). Both roscovitine and M3 also reduced *B. cenocepacia* burden in non-CF MDMs (Fig. 2A,B). We then examined roscovitine and M3 compared to and in combination with the compound cysteamine, as we recently demonstrated that cysteamine increased macrophage-mediated killing of common CF pathogens^22^. Cysteamine alone demonstrated a similar reduction in *B. cenocepacia* load in CF MDMs compared to 50 μM roscovitine (Fig. 2A) and M3 (Fig. 2B). However, there was an increased dose-dependent reduction in bacterial load in CF MDMs when cysteamine was combined with increasing concentrations of roscovitine (Fig. 2A) or M3 (Fig. 2B). When cysteamine was combined with 50 μM roscovitine or M3 there was a 4 to 5 log reduction in bacterial load. A similar trend was observed in non-CF MDMs.

Next, we examined the capacity of CF MDMs treated with roscovitine or M3 to kill a multi-drug resistant clinical strain of *P. aeruginosa* isolated from a clinic patient. Roscovitine (Fig. 2C) and M3 (Fig. 2D) both demonstrated a dose-dependent reduction in *P. aeruginosa* bacterial load in CF MDMs, similar to *B. cenocepacia*. However, there was no change in bacterial load in non-CF MDMs for roscovitine, but a significant decrease with M3. In contrast to *B. cenocepacia*, treatment of *P. aeruginosa*-infected MDMs with cysteamine alone was equivalent to 5–20 μM concentrations of roscovitine (Fig. 2C) or M3 (Fig. 2D). However, we did observe an increased effect of cysteamine on *P. aeruginosa* bacterial load when combined with roscovitine (Fig. 2C) or M3 (Fig. 2D) in both CF and non-CF MDMs.

**Figure 3:**
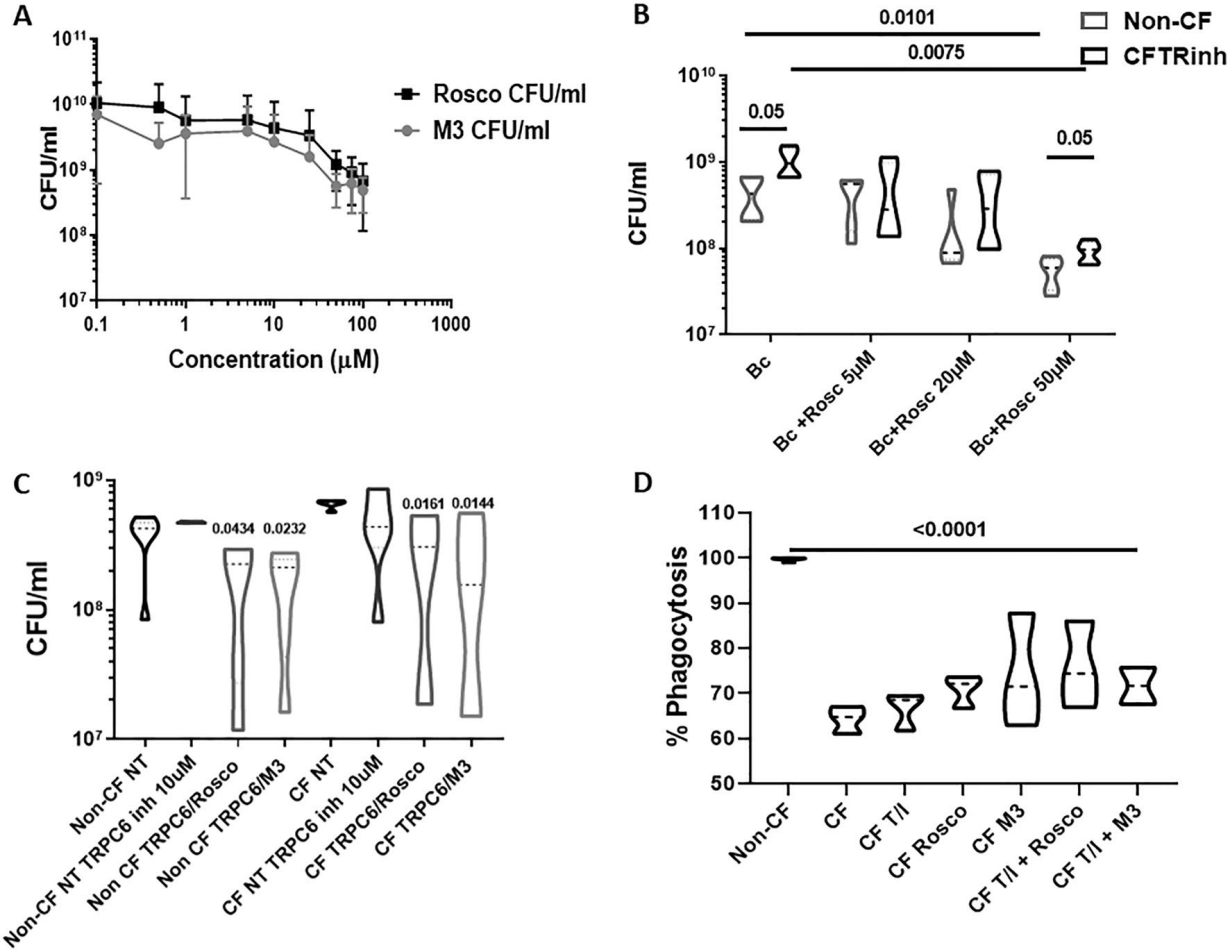
Roscovitine’s impact on killing of bacteria is partially TRPC6 and CFTR-dependent. **(A)** CFUs for 24 h bacterial killing assay of roscovitine or M3 against multi-drug resistant *B. cenocepacia* (Bc) in media devoid of human cells. n = 4, significance via t-test. B) CFU assay for non-CF MDMs infected with *B. cenocepacia* in the presence (CFTRinh) or absence (non-CF) of a CFTR inhibitor and increasing concentrations of roscovitine. n = 5, significance via t-test with group comparison via one-way ANOVA. Group comparisons to Bc alone. Group ANOVA p value 0.004. Data represented as violin plots to show distribution of data **(C)** CFU assay for CF and non-CF MDMs infected with *B. cenocepacia* in the absence (NT) or presence of 10 μM TRPC6 inhibitor for 4 h, with or without 50 μM roscovitine or 50 μM M3. n = 5. Significance via t-test with group comparison via one-way ANOVA. Group ANOVA p value 0.0019. Group comparisons to non-CF NT and CF NT. Data represented as violin plots to show distribution of data. D) Summed CF MDM % phagocytosis of RFP-expressing *B. cenocepacia* normalized to non-CF MDMs, with or without treatment with tezacaftor/ivacaftor (T/I), roscovitine, M3 or combinations. n = 4, MOI 50, one-way ANOVA with post-hoc Tukey p value < 0.0001.

Because of the observed effects of roscovitine and M3 on macrophage-mediated bacterial killing, we tested the ability of roscovitine and M3 to kill *B. cenocepacia* in the absence of macrophages. In a direct killing assay, we saw a similar impact of roscovitine and M3 upon *B. cenocepacia* bacterial load, with a dose-dependent decrease in bacterial load with increasing concentrations up to 100 μM (Fig. 3A). A direct reduction in bacterial load was not achieved until concentrations of 50 μM (Fig. 3A).

### Roscovitine’s impact on killing of bacteria is partially dependent on CFTR and TRPC6

Roscovitine has broad mechanistic actions, including stabilization of CFTR and indirect activation of the TRPC6 Ca^2+^ channel^14, 15^. To further test the role of roscovitine when CFTR is present but functionally inhibited, we isolated non-CF MDMs and infected them with *B. cenocepacia* in the presence or absence of a CFTR inhibitor and increasing concentrations of roscovitine. CFTR inhibition alone increased *B. cenocepacia* bacterial load compared to uninhibited non-CF MDMs (Fig. 3B). Treatment with 50 μM roscovitine significantly reduced bacterial load in non-CF MDMs (Fig. 3B). Roscovitine treatment of CFTR inhibited MDMs also recapitulated our prior finding of reduced bacterial load in primary CF MDMs (**Fig. ****3****B**). A significant difference in bacterial load between non-CF MDMs and CFTR-inhibited MDMs persisted during roscovitine treatment (Fig. 3B). These results suggest a partial dependence on CFTR for roscovitine-mediated bacterial killing.

To test the role of the TRPC6 channel in CF MDMs during roscovitine treatment, non-CF and primary CF MDMs were infected with *B. cenocepacia* in the presence or absence of a TRPC6 inhibitor ^17^ (10 µM, 4 h) and/or roscovitine or M3. TRPC6 channel inhibition alone did not directly impact bacterial load in either CF or non-CF MDMs (Fig. 3C). However, there was a significant reduction in bacterial load for both CF and non-CF MDMs when TRPC6-inhibited MDMs were treated with either roscovitine or M3 (Fig. 3C). The reduced bacterial load during roscovitine/M3 treatment and TRPC6 inhibition was attenuated in magnitude when compared to Fig. 2 results for roscovitine or M3 alone. These results suggest that roscovitine-mediated bacterial killing is partially dependent on TRPC6.

### Roscovitine enhances CFTR modulator-induced functional changes in macrophages

We recently demonstrated that CF MDMs have defective bacterial phagocytosis, which can be partially rescued by administration of a CFTR modulator^20^. To determine if roscovitine or M3 enhanced killing of bacteria via improved phagocytosis, we measured phagocytosis of *B. cenocepacia* in CF MDMs in the presence or absence of roscovitine, M3, or the CFTR modulator tezacaftor/ivacaftor. Untreated CF MDMs demonstrated a significant reduction in phagocytosis at baseline compared to non-CF (**Fig. ****3****D**), recapitulating our prior findings^20^. There was no improvement in CF phagocytosis with the addition of tezacaftor/ivacaftor, roscovitine, or M3 alone (Fig. 3D). There was no significant improvement in phagocytosis when tezacaftor/ivacaftor was combined with either roscovitine or M3 (Fig. 3D). These results suggest that roscovitine does not exert its primary effects through enhanced bacterial phagocytosis.

**Figure 4:**
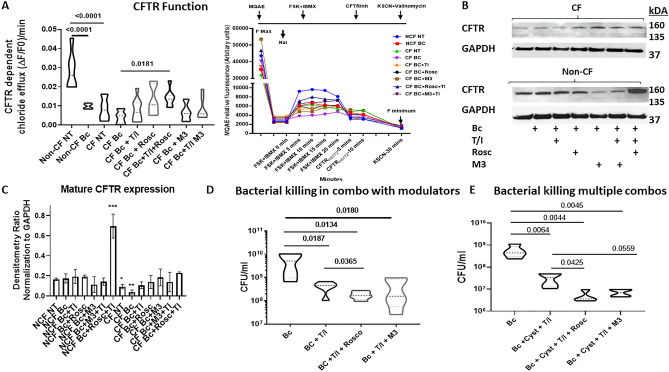
Roscovitine enhances CFTR modulator-induced functional changes in macrophages. Data represented as violin plots to show distribution of data. All this figure experiments utilized 50 μM roscovitine or M3. **(A)** Halide efflux assay for CFTR function in non-CF and CF MDMs without (NT) or with infection with *B. cenocepacia* (Bc). CF MDMs were additionally treated with tezacaftor/ivacaftor (T/I), roscovitine (Rosc), M3, or T/I combined with roscovitine or M3. Results are presented as summary of mean CFTR function at peak of forskolin stimulation (10 min) as well as in a corresponding chloride efflux time course. n = 6–9, one-way ANOVA with post-hoc Tukey. Group ANOVA p value < 0.0001. **(B)** Representative western blot for CFTR in CF and non-CF MDMs infected with *B. cenocepacia *(*Bc*) and treated with tezacaftor/ivacaftor (T/I) roscovitine, (Rosc), M3, or T/I combined with roscovitine or M3. **(C)** Densitometric analysis of **(B)** normalized to loading control GAPDH, n = 3, significance determined relative to non-CF NT. * = p value < 0.05, ** = p value < 0.01., *** = p value < 0.001. **(D)** CFU assay for CF MDMs infected with *B. cenocepacia *(*Bc)* and treated with tezacaftor/ivacaftor (T/I) or T/I combined with roscovitine or M3. n = 5. Group ANOVA p value 0.0084. **(E)** CFU assay for CF MDMs infected with *B. cenocepacia (Bc*) and treated with tezacaftor/ivacaftor (T/I) and cysteamine, or T/I and cysteamine combined with roscovitine or M3. n = 5. Group ANOVA p value 0.0015.

Next, we measured changes in CFTR-dependent chloride efflux in CF and non-CF MDMs as a surrogate of CFTR function. MDMs were infected with *B. cenocepacia* in the presence or absence of tezacaftor/ivacaftor, roscovitine, M3 or combinations. Using a halide efflux assay, we found that CF MDMs had significantly reduced CFTR function at baseline compared to non-CF MDMs (Fig. 4A). Infection with *B. cenocepacia* significantly reduced CFTR function in non-CF MDMs and further reduced CFTR function in CF MDMs (Fig. 4A). Treatment of *B. cenocepacia*-infected CF MDMs with tezacaftor/ivacaftor or roscovitine alone led to increases in CFTR function up to the level observed in infected non-CF MDMs. Only a combination of tezacaftor/ivacaftor and roscovitine significantly increased CFTR function in CF MDMs during infection compared to no treatment (Fig. 4A). In contrast to roscovitine plus tezacaftor/ivacaftor, the combination of M3 and tezacaftor/ivacaftor did not change CFTR function compared to tezacaftor/ivacaftor alone (Fig. 4A).

To determine if CFTR expression was also impacted by infections or treatments, we measured CFTR protein expression during infection with *B. cenocepacia* in the presence or absence of tezacaftor/ivacaftor, roscovitine, M3, or combinations. We found that infection alone was not associated with changes in CFTR protein expression by western blot in CF or non-CF MDMs (Fig. 4B,C). Treatment with roscovitine alone, roscovitine plus tezacaftor/ivacaftor, M3 alone or M3 plus tezacaftor/ivacaftor all were associated with a trend towards increased CFTR expression in CF MDMs during infection, but variability was noted within donors (Fig. 4B,C). Interestingly, treatment with M3 decreased CFTR expression in some non-CF donors and the combination of roscovitine plus tezacaftor/ivacaftor significantly increased CFTR expression in non-CF (Fig. 4B,C). Examples of uncropped western blots for Fig. 4 can be found in Supplemental Fig. 1.

We then performed a CFU assay in CF MDMs during infection with *B. cenocepacia* in the presence of tezacaftor/ivacaftor with or without roscovitine or M3. CF MDMs treated with tezacaftor/ivacaftor alone had a significant reduction in bacterial burden (Fig. 4D). However, the combination of roscovitine and tezacaftor/ivacaftor further reduced bacterial burden compared to tezacaftor/ivacaftor alone (Fig. 4D). Similar to our CFTR functional results, the combination of M3 and tezacaftor/ivacaftor did not change bacterial burden compared to tezacaftor/ivacaftor alone (Fig. 4D).

Last, because we had previously shown that cysteamine combined with roscovitine had additive effects on killing of *B. cenocepacia*, we performed a CFU assay for combinations of roscovitine/M3, tezacaftor/ivacaftor, and cysteamine. Cysteamine treatment combined with tezacaftor/ivacaftor significantly reduced bacterial burden compared to infection alone (Fig. 4E). The addition of roscovitine but not M3 to cysteamine/tezacaftor/ivacaftor led to a further reduction in bacterial burden by at least 1 log compared to the cysteamine/tezacaftor/ivacaftor combination alone (Fig. 4E).

Combined, our data suggest efficacy of roscovitine-mediated killing of resistant CF pathogens, but the greatest efficacy is achieved when a multi-pronged approach is utilized with CFTR modulators and cysteamine.

## Discussion

Treatment of multi-drug resistant bacterial infections remains an important clinical problem in many diseases, including CF. In particular, *Burkholderia* infections in CF are extremely difficult to treat, lacking standardized antibiotic regimens^28^, an exclusion to lung transplantation and often from clinical trials testing new anti-bacterial products. Furthermore, there is a growing incidence of *Burkholderia cepacia* complex healthcare-acquired infections in patients without CF^29, 30^. To this end, we demonstrated the ability of roscovitine to enhance macrophage-mediated killing of *Burkholderia cenocepacia* as well as multi-drug resistant *Pseudomonas aeruginosa*. Roscovitine also showed enhanced efficacy in combination with other existing therapeutics such as CFTR modulators and cysteamine. Our findings have important implications for future therapeutic regimens in CF.

Although we demonstrated efficacy of roscovitine alone or in combination with the CFTR modulator tezacaftor/ivacaftor, the strongest effect of roscovitine was in combination with the transglutaminase 2 inhibitor cysteamine. Previously we demonstrated that cysteamine reduces transglutaminase 2 accumulation in CF^22^, leading to increased *Burkholderia* uptake into autophagosomes and subsequent improved clearance. In our current study we showed that roscovitine has a similar increase in killing of *B. cenocepacia* compared to cysteamine, but combined they reduce bacterial burden by 4–5 log. In contrast, we found more incremental additive effects of roscovitine and cysteamine when macrophages were infected with *P. aeruginosa.* However, CF macrophages were more easily able to control *P. aeruginosa* burden, with lower starting and treatment-related bacterial burden compared to *B. cenocepacia*. These results suggest that pathogen specific effects (such as subversion of autophagy for *B. cenocepacia*) may be important in dictating which bacteria require a combinatorial treatment approach, compared to solitary treatment with roscovitine. Further, we do not know if the observed reductions in *B. cenocepacia* with roscovitine treatment alone would be clinically significant.

In addition to an impact upon killing of bacteria, we found that roscovitine increases CFTR function in primary CF macrophages, particularly when combined with the CFTR modulator tezacaftor/ivacaftor. Roscovitine has known effects on CFTR, having been shown to inhibit CFTR degradation by proteases and subsequently lead to increased CFTR trafficking to the plasma membrane and partial restoration of CFTR function^15^. Roscovitine-mediated increases in macrophage CFTR function mirrored changes in killing of *B. cenocepacia*, with a greater reduction in bacterial burden when roscovitine was combined with tezacaftor/ivacaftor compared to tezacaftor/ivacaftor alone. Our results further support the concept that roscovitine could have pleiotropic effects in CF through restoration of CFTR function. Further work will be needed to determine how roscovitine interacts with emerging CFTR modulator combinations such as tezacaftor/ivacaftor/elexacaftor^31^, which have shown improved clinical benefits compared to tezacaftor/ivacaftor.

Despite a relatively good tolerance, roscovitine did not demonstrate efficacy in the ROSCO-CF clinical trial (Meijer et al., unpublished). There are many possible reasons that could explain the discrepancy between the encouraging results obtained with roscovitine on various cell^14, 15^ and animal (Bonfield T et al., unpublished) models of CF and the disappointing lack of beneficial effects of roscovitine in the clinic. Some of these reasons may be the short duration of the trial, the low number and biological diversity of patients, and inappropriate dosing (dose, formulation, administration mode, frequency of administration and timing [circadian rhythm was not taken into account]). Another reason may stem from the fact that roscovitine was not optimized for CF, and just re-purposed from anticancer activity to the CF pathology. Classical medicinal chemistry guided optimization starting from roscovitine and M3 should lead to a drug candidate with much improved efficacy. In addition, this optimization should be carried out in a combination approach with established CFTR modulators, as clearly encouraged by the results presented here. Finally, it is possible that the strong anti-inflammatory effects or roscovitine^13^ could have a negative impact on host responses to chronic infection in CF, and negate potential benefits mediated by enhanced killing of bacteria. We believe that this possibility is less likely given roscovitine’s overall safety profile, success in CF pre-clinical studies, and benefits of other anti-inflammatories in CF^16^ such as azithromycin or ibuprofen. Never-the-less, changes in inflammatory responses during future trials of roscovitine or metabolites are an important design consideration.

In summary, our data support the use of roscovitine or derivatives of it for multi-drug resistant bacterial infections in CF. Roscovitine enhances macrophage function including CFTR-mediated ion efflux and has additive benefits when combined with existing CFTR modulators or cysteamine. Further clinical trial testing of roscovitine and optimized derivatives and analogues in CF is warranted.

## Supplementary Information

Supplementary Figure 1.

## Data Availability

Data are available upon request to the authors.

## Abbreviations

CF: Cystic fibrosis
CFTR: Cystic fibrosis transmembrane conductance regulator
CFU: Colony-forming unit assay
IBMX: 3-Isobutyl-1-methylxanthine
MDM: Monocyte-derived macrophage
MQAE: *N*-[Ethoxycarbonylmethyl]-6-methoxy-quinolinium bromide
