# Supplementary material for: (R)-Roscovitine and CFTR modulators enhance killing of multi-drug resistant Burkholderia cenocepacia by cystic fibrosis macrophages
Source: Sci Rep. 2020 Dec 10;10:21700. doi: 10.1038/s41598-020-78817-x (PMC7728753; doi:10.1038/s41598-020-78817-x)
Supplement: Supplementary file 1 — Supplementary Figure 1. [file 41598_2020_78817_MOESM1_ESM.docx]

**SUPPLEMENTAL INFORMATION**

**(R)-ROSCOVITINE AND CFTR MODULATORS ENHANCE KILLING OF MULTI-DRUG RESISTANT *BURKHOLDERIA CENOCEPACIA* BY CYSTIC FIBROSIS MACROPHAGES**

Running title: Roscovitine and *Burkholderia*

Chandra L. Shrestha^1^, Shuzhong Zhang^1^, Benjamin Wisniewski^1^, Stephanie Häfner ^2^, Jonathan Elie^3^, Laurent Meijer^3^, Benjamin T. Kopp^1*^

^1^*Center for Microbial Pathogenesis, The Abigail Wexner Research Institute at Nationwide Children’s Hospital, Columbus, OH, USA*

*^2^ Rudolf-Boehm-Institut f. Pharmakologie u. Toxikologie Medizinische Fakultät, Universität Leipzig, Leipzig, Germany*

*^3^ManRos Therapeutics, Perharidy Peninsula, Roscoff, France*

*To whom correspondence should be addressed:

Benjamin Kopp

Nationwide Children's Hospital

Division of Pulmonary Medicine

700 Children’s Drive

Columbus, OH 43205

tel. 614-722-4766; fax 614-722-4755

e-mail: Benjamin.Kopp@NationwideChildrens.org

Keywords: Cystic fibrosis, macrophage, therapeutics, *Burkholderia,* roscovitine

Portions of this manuscript were presented at the 2019 North American Cystic Fibrosis Conference.

**Supplemental Figure 1: Uncropped western blots from Figure 4B**

**CF CFTR CFFT antibody CF GAPDH Non-CF CFTR CFFT Non-CF GAPDH
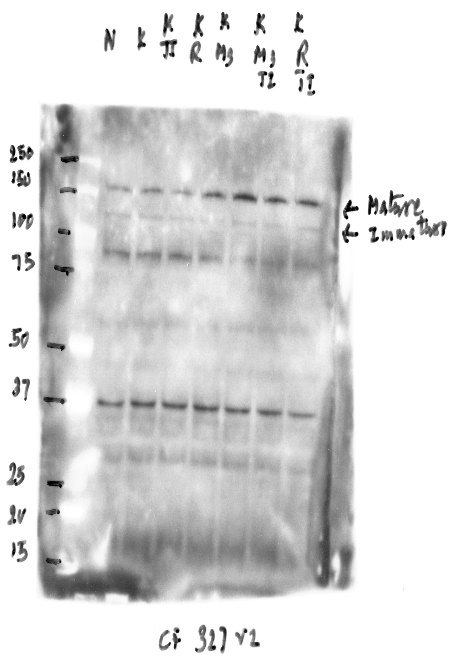
** **
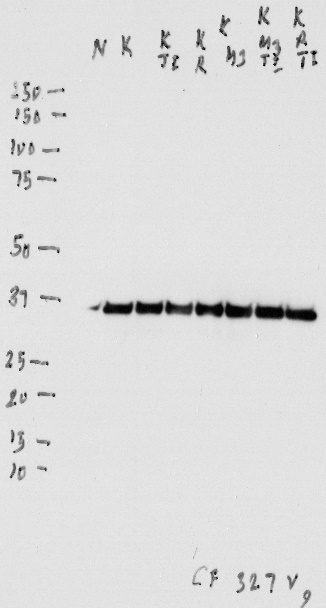

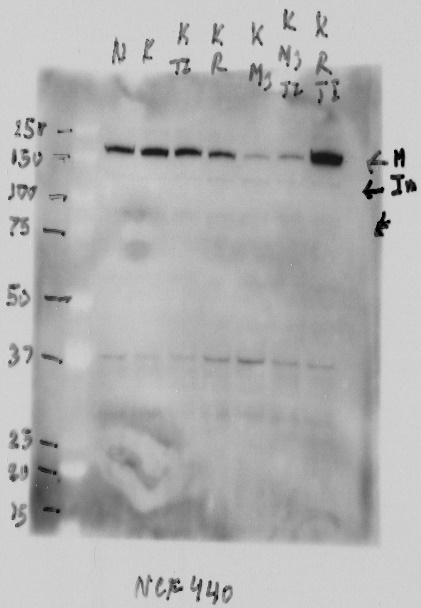

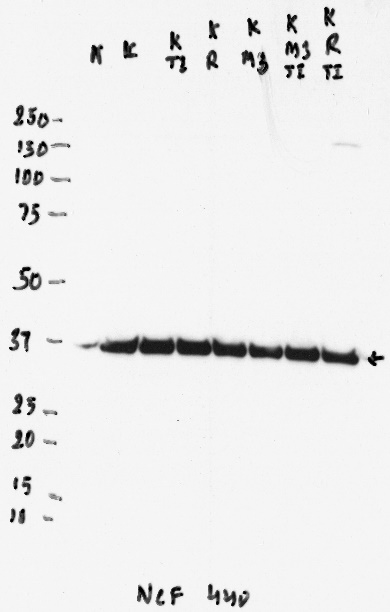
**

**CF CFTR CFFT antibody CF GAPDH Non-CF CFTR CFFT Non-CF GAPDH**

**
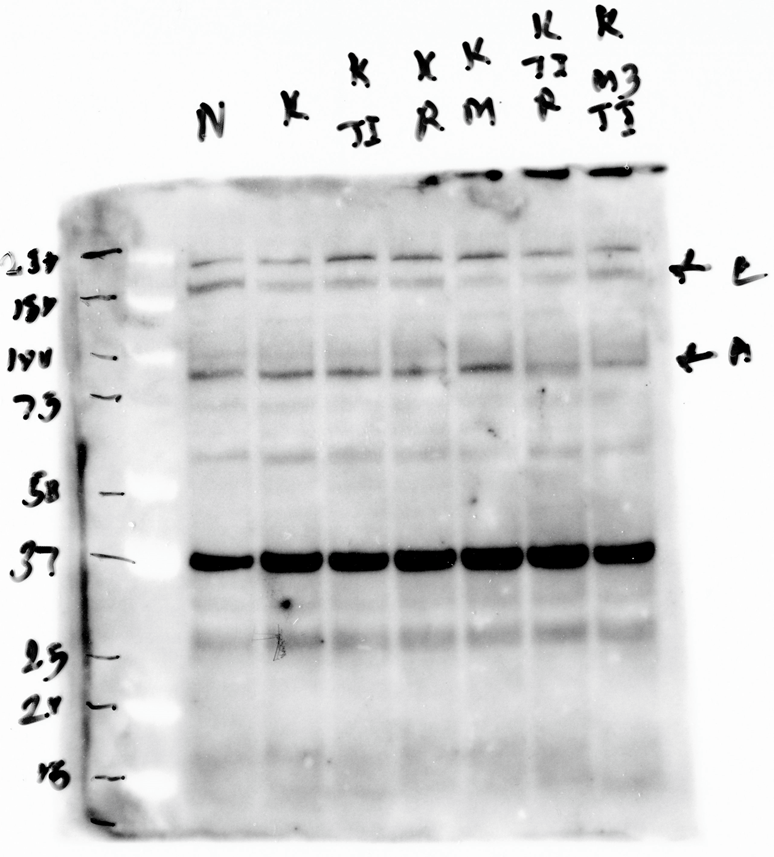

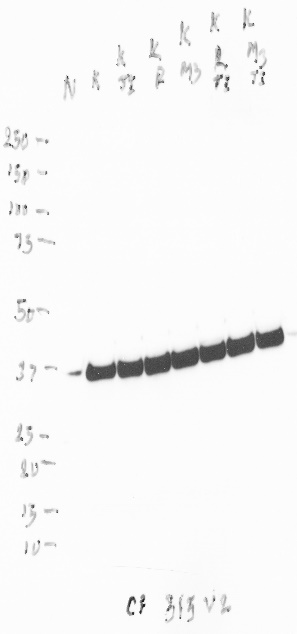

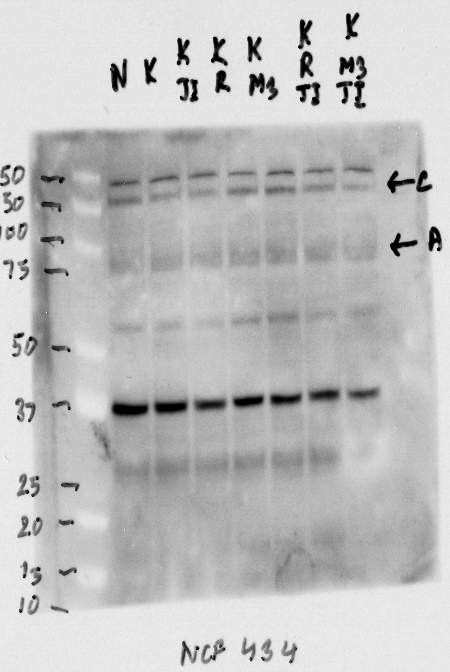

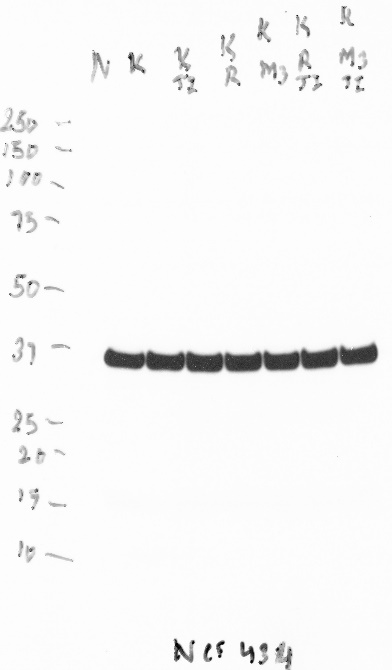
**
